# *ESR1* PvuII polymorphism: from risk factor to prognostic and predictive factor of the success of primary systemic therapy in advanced breast cancer

**DOI:** 10.1186/s12885-021-09083-x

**Published:** 2021-12-20

**Authors:** Ramadhan Karsono, Samuel J. Haryono, Bambang Karsono, Wirsma Arif Harahap, Yulia Pratiwi, Teguh Aryandono

**Affiliations:** 1 Department of Surgical Oncology, Dharmais Hospital-National Cancer Center, Jakarta, Indonesia; 2 Department of Hematology and Medical Oncology, Dharmais Hospital-National Cancer Center, Jakarta, Indonesia; 3 Surgical Oncology Division, Faculty of Medicine Universitas Andalas/Dr. M Djamil General Hospital Padang, West Sumatera, Indonesia; 4 Functional Medical Staff of Surgical Oncology Department, Dharmais Hospital-National Cancer Center, Jakarta, Indonesia; 5 Department of Surgery, Faculty of Medicine Public Health and Nursing, Universitas Gadjah Mada, Yogyakarta, Indonesia

**Keywords:** ESR1 PvuII, Breast cancer, Hormonal, Chemotherapy, Indonesia

## Abstract

**Background:**

The *ESR1* gene encodes Estrogen Receptor alpha (ERα), which plays a role in the tumourigenesis of breast cancer. A single nucleotide polymorphism (SNP) in intron 1 of this gene called *ESR1* PvuII (rs2234693) has been reported to increase the risk of breast cancer. This study aimed to investigate the *ESR1* PvuII polymorphism as a prognostic and predictive factor guiding the choice of therapy for advanced breast cancer.

**Methods:**

This retrospective study was conducted in 104 advanced breast cancer patients at Dharmais Cancer Hospital from 2011 to 2018. The *ESR1* PvuII polymorphism was analysed by Sanger sequencing of DNA from primary breast tumour samples.

**Results:**

The percentages of patients with *ESR1* PvuII genotypes TT, TC, and CC were 42.3, 39.4, and 18.3%, respectively. Looking at prognosis, patients with *ESR1* PvuII TC + CC had shorter overall survival than those with the TT genotype [HR = 1.79; 95% CI 1.05–3.04; *p* = 0.032]. As a predictive marker, TC + CC was associated with shorter survival (*p* = 0.041), but TC + CC patients on primary hormonal therapy had a median overall survival longer than TC + CC patients on primary chemotherapy (1072 vs 599 days).

**Conclusion:**

The *ESR1* PvuII TC + CC genotypes confer poor prognosis in advanced breast cancer, but these genotypes could be regarded as a good predictor of the therapeutic effect of hormonal treatment.

## Background

Breast cancer is the most common cancer in women and is a heterogeneous disease based on several molecular subtypes by immunohistochemistry, epidemiological risk, and response to treatment [1]. In each individual with breast cancer, there is a set of genetic aberrations that can be informative in identifying their risk, choosing their therapy, and making a prognosis. Information on genetic aberrations in cancer will lead to more precise treatments [2].

Over two-thirds of breast cancers express estrogen receptor α protein (encoded by *ESR1*) which plays a role in the tumourigenesis of breast cancer [3, 4]. Recent, retrospective analyses of *ESR1* mutations in circulating tumour DNA suggested that the occurrence of the mutations was associated with poor overall survival and resistance to hormonal treatment in patients with metastatic disease [5]. The majority of these mutations are located in exon 8, within the ligand-binding domain (LBD), and create a ligand-free constitutively activated ER, which has been associated with a worse outcome and could be considered a predictive marker guiding therapeutic decision making [6, 7].

An intronic polymorphism in the *ESR1* gene (rs2234693), also called *ESR1* PvuII, is associated with an increased risk of breast cancer and decreased estrogen receptor (ER) expression [3]^.^ Recent data from several studies have garnered interest in investigating the potential role of *ESR1* mutational status as a predictive marker and a tool to guide clinicians in choosing therapies, but there are many limitations to developing predictive biomarkers [6]. In this study, we investigated *ESR1* PvuII as a prognostic and predictive factor for the selection of therapy in advanced breast cancer.

## Methods

### Study design and patients

This was a retrospective study that included 104 consecutive advanced breast cancer patients who had been treated between 2011 and 2018 in Dharmais Cancer Center Hospital. Advanced breast cancer included both locally advanced disease (stage 3B and 3C) and metastatic breast cancer with distant metastases to other organs, commonly the skeleton, lung, brain, and liver [8]. Patients were included who met the inclusion criteria and had complete data on both tissue characteristics and follow-up status. Fresh tissue was taken before primary systemic treatment. Patients with complete treatment were those who were given primary hormonal therapy for 6 months or primary chemotherapy within 6 cycles.

Therapeutic options were chosen based on the treatment protocol in the NCCN guidelines [9]. The agents available to the primary hormonal therapy group were Aromatase Inhibitor (AI) and Tamoxifen for postmenopausal patients and premenopausal patients, respectively. The patients, received Tamoxifen only or bilateral salpingo-oophorectomy (BSO) plus AI/Tamoxifen, or if patients rejected BSO they were given Gonadotropin-Releasing Hormone Analogue (GnRHa) and AI/Tamoxifen for 6 months. The AI was Letrozole, Anastrozole, or Exemenestane.

The primary chemotherapy group received FAC (5-Fluorouracil, Adriamycin, and Cyclophosphamide) which was given for 6 cycles. In this study, patients with HER2+ cancer did not receive anti-HER2 agents. The patients provided written informed consent to participate in the study, which was approved by the Ethics Committee of Dharmais Hospital (ethical clearance numbers 049/PEP/08/2011 and 199/KEPK/XI/2019).

### Mutational analysis

**Fig. 1:**
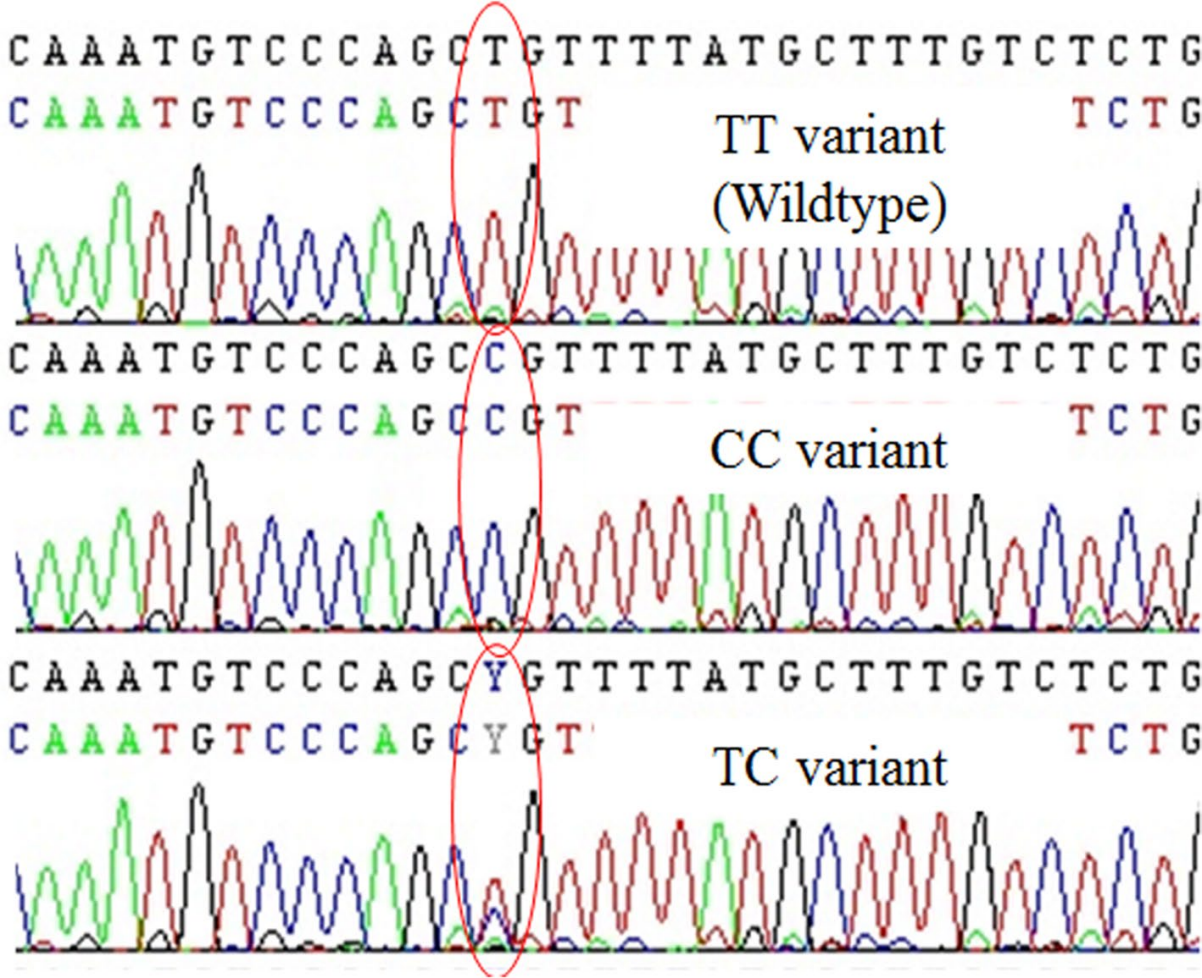
The result of Sanger sequencing showing a different type of ESR1 PvuII Polymorphism. The red circle show the base change (Y is bioinformatics code for the presence of T or C)

DNA samples from primary breast tumours were processed by Polymerase Chain Reaction (PCR) using the *ESR1* forward primer TGT AAA ACG ACG GCC AGT TCA CGC AGT CTG GAG TTG TC and reverse primer CAG GAA ACA GCT ATG ACC AGA CCA ATG CTC ATC CCA AC. The total product was 519 bp, which was sequenced by Sanger sequencing with BigDye v3.1 reagent [Applied Biosystems]. Sequence data were analysed using Bioinformatics Software [Seqscape] and combined with clinicopathological data. The sequenced of *ESR1* PvuII Polymorphism (Fig. 1) was divided into wild-type (TT variant), TC variant, and CC variant.

### Statistical analysis

Statistical analysis was performed using IBM SPSS 21. Associations between *ESR1* PvuII polymorphism and clinicopathological variables were assessed by the chi-square test (χ^2^ test). All analyses were hypothesis-driven, and *P* < 0.05 was considered statistically significant. Overall survival (OS) was defined as the time from diagnosis until death from any cause. OS rates were estimated using the Kaplan-Meier method. A Cox proportional hazards model was used to estimate the prognostic value of *ESR1* PvuII Polymorphism on overall survival (OS). To estimate the predictive factor of genotypes on OS, hazard ratios (HRs) with 95% confidence interval (CIs) were calculated for primary hormonal therapy vs. primary chemotherapy in the TT and TC + CC groups.

## Results

### Correlation of *ESR1* PvuII genotype with survival

#### Correlations type of *ESR1* PvuII polymorphism with treatment effect and survival

In the TT group, patients who underwent primary hormonal therapy had a median OS of 1375 days (95% CI, 983–1766 days) compared with 951 days for patients who underwent primary chemotherapy (Fig. 3A). There was a significant difference in survival in the TC + CC variant group (Fig. 3B), as these patients survived longer after primary hormonal therapy than primary chemotherapy (1072 vs. 599 days).

## Discussion

Breast cancer in Indonesia, as in other developing countries, is disregarded and mostly diagnosed late, at stages 3 and 4, at which time the patient has a low life expectancy [10]. The persisting issue in a clinical setting of advanced breast cancer is the type of therapy give. Currently, oncologists routinely apply the clinical TNM staging system and detect the ER, progesterone receptor (PR), and Her2 proteins in the tumour cells [9]. Most clinicians tend to give chemotherapy as soon as possible and ignore the ER/PR as the hormonal status, whose expression remains the lead function in ER/PR positive cases, and but this approach seems to give no extra consideration to the underlying obstacles and benefits [11]. The Cochrane meta-analysis for advanced breast cancer showed no difference in survival between chemotherapy or hormonal therapy and chemotherapy worsened quality of life [12]. Our study found that there was a statistically significant difference survival between patients given hormonal therapy and patients given chemotherapy in the TC + CC genotype group, where hormonal therapy yielded a longer survival than chemotherapy.

Only few prospective studies have compared primary chemotherapy with primary hormonal therapy in advanced breast cancer [12–17]. Treatment selection of advanced breast cancer is based on hormonal receptors at the protein levels rather than at the genotype level, according to the NCCN guidelines HR-positive patients will be given hormonal therapy whereas HR-negative or Her2+ and visceral metastasis patients will receive chemotherapy [9]. Chemotherapy for advanced stages hormone receptor-positive cases breast cancer with visceral metastasis and Her2+ positivity does not prolong life expectancy [12]. Severe side effects of chemotherapy have become a reason to minimize the administration this treatment to patients. A new parameter is needed that can predict which patients should receive chemotherapy or hormonal therapy.

*ESR1* SNPs are associated with tumour carcinogenesis, cell proliferation, metastasis, and prognostic [18–20]. Every woman with breast cancer has the *ESR1* gene but in only 70–80% of breast tumours is ERα expressed, as shown by immunohistochemistry [21]. Several mechanisms have been shown to silence ER expression, such as *ESR1* mutations, polymorphisms, epigenetic events, and posttranslational modification events [22, 23]. Immunohistochemical detection of hormone receptor expression is often a problem in clinical practice (Table 1) [24].

**Table 1:** Correlations between *ESR1* PvuII polymorphisms and clinicopathological features

Characteristics	***ESR1*** PvuII Polymorphism		***P***-value
	TT genotype (***n*** = 44)	TC + CC genotype (***n*** = 60)	
**Age at biopsy**			
Mean (+ SD)	47,5 (9,5)	48,1 (10,9)	0,779^b^
Median (range)	48,5 (28–68)	47 (22–75)	
**Grade**			
Low	19 (36,5)	33 (63,5)	0,234^a^
High	25 (48,1)	27 (51,9)	
**Hormonal Receptor**			
Negative	9 (34,6)	17 (65,4)	0,359^a^
Positive	35 (44,9)	43 (55,1)	
**ER (Estrogen Receptor)**			
Negative	13 (40,6)	19 (59,4)	0,817^a^
Positive	31 (43,1)	41(56,9)	
**PR (Progesteron Receptor)**			
Negative	13 (41,9)	18 (58,1)	0,96^a^
Positive	31 (42,5)	42 (57,5)	
**Her2 status**			
Negative	33 (44,6)	41 (55,4)	0,458^a^
Positive	11 (36,7)	19 (63,3)	
**Histology**			
Ductal	41 (43,6)	53 (56,4)	0,407^a^
Lobular	3 (30,0)	7 (70,0)	
**Therapy**			
Primary hormonal therapy	24 (54,5)	29 (48,3)	0,531^a^
Primary chemotherapy	20 (45,5)	31 (51,7)	

*P* value: ^a^ = Pearson Chi Square; ^b^ = Independent sample T test

In general ESR1 PvuII (rs2234693) changes the proteins detectable in blood and has been used as an inherited risk factor for Asian people [25]. This paper does not discuss breast cancer risk factors, but we will discuss further how this SNP is present in tumour tissue and can be used as a predictor of the best therapy. This translational study is the first analysis of a novel genetic predictor that could help us choose between chemotherapy or hormonal therapy as the primary treatment for advanced breast cancer.

**Fig. 2:**
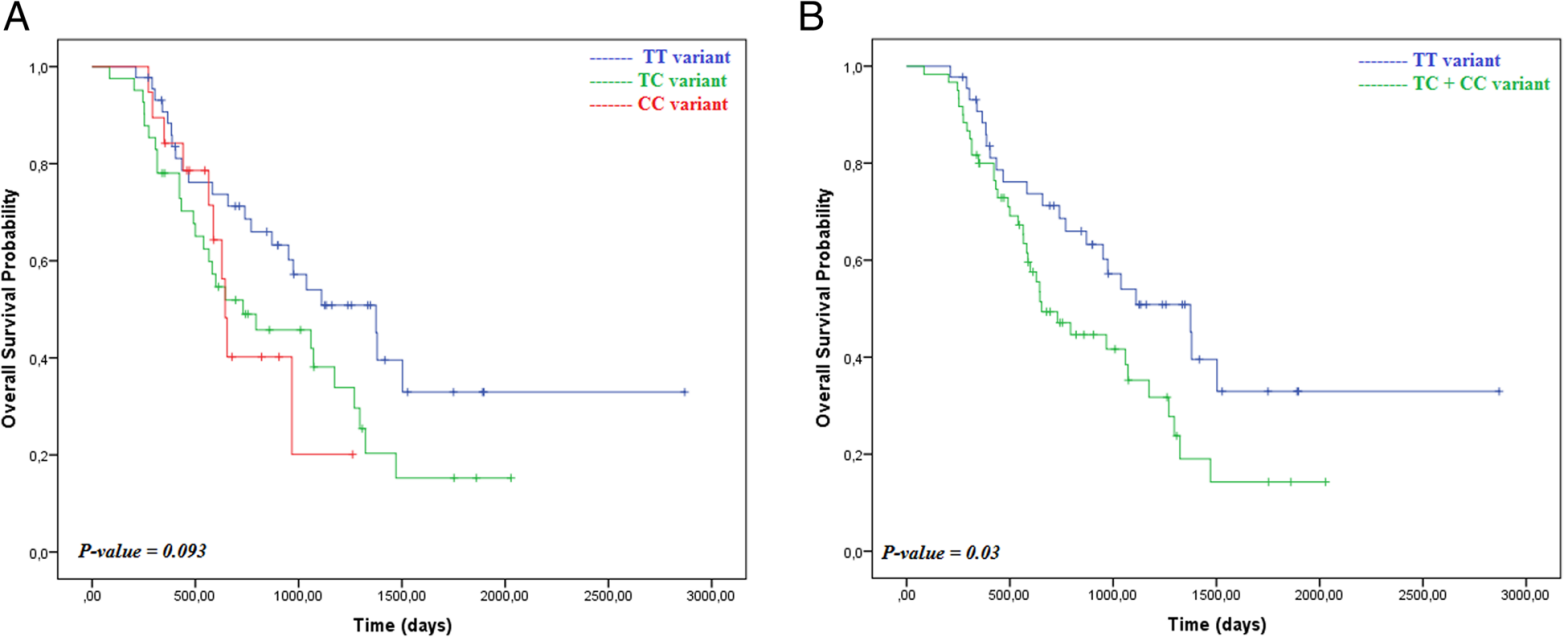
Kaplan-Meier curves for Overall Survival (OS) according to ESR1 PvuII Polymorphism genotypes. Comparing overall survival between all three genotypes individually (**A**), Probability of Overall Survival for TT vs TC + CC variants (**B**)

As a prognostic factor, the TT genotype was correlated with longer survival than the TC and CC genotypes (Fig. 2A & B). The risk of death in the TC + CC genotype group was higher than that in the TT genotype group, and the highest risk of death was in the CC genotype subgroup (Table 2). Blood detectable *ESR1* mutations in exon 8 after AI failure have been associated with a worse prognosis for overall survival than wild-type *ESR1* [6, 26].

**Table 2:** Overall Survival by *ESR1* PvuII polymorphism alleles

Group	No.	Events	OS, Median (95% CI), days	Hazard Ratio (95% CI)	***P***-value
TT variant	44	22	1375 (965–1784)	NA	
TC variant	41	28	730 (237–1222)	1.77 (1.01–3.1)	0.046
CC variant	19	10	644 (601–686)	1.85 (0.85–4.01)	0.117
TC + CC variants	60	34	654 (449–858)	1.79 (1.05–3.04)	0.032

Abbreviation: *NA* not applicable

**Fig 3:**
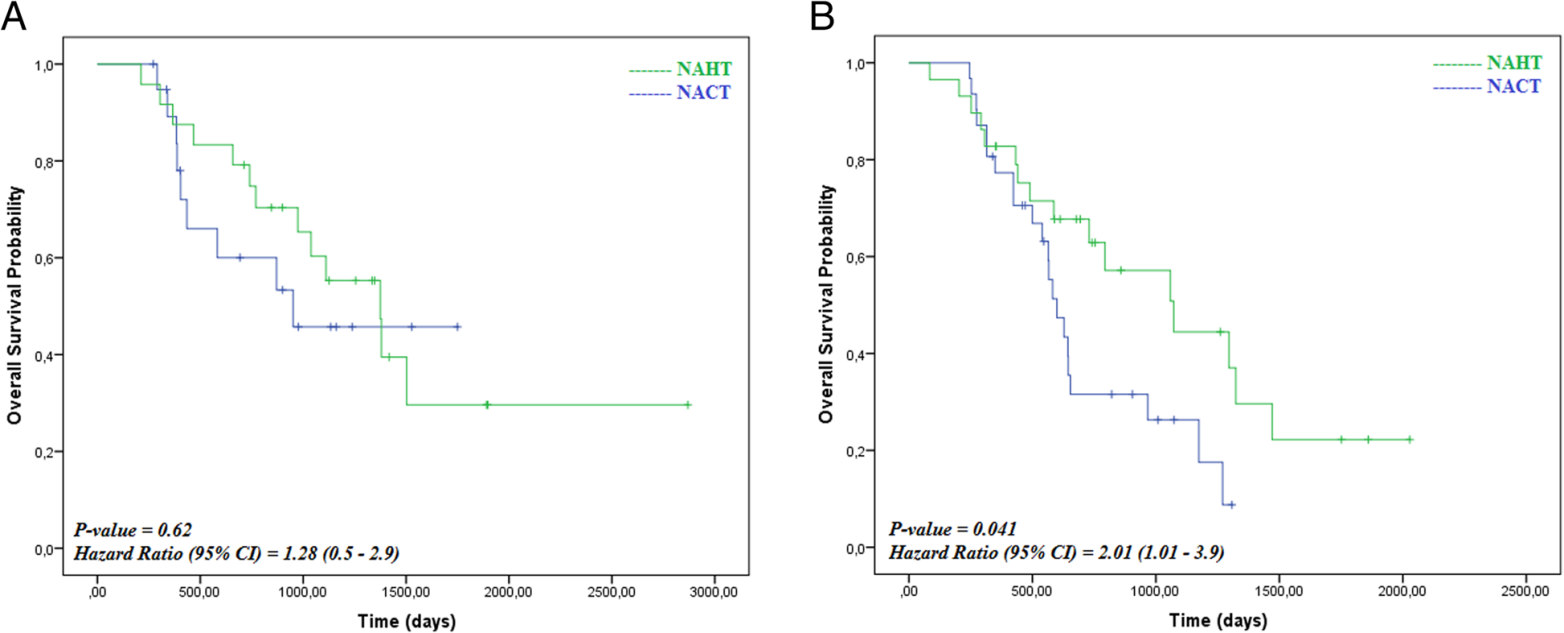
Kaplan-Meier Curves for Overall Survival (OS) According to Therapy by *ESR1* Polymorphism Status. Probability of Overall Survival in TT group (A); in TC+CC group (B)

This study found a statistically significant difference between the survival of patients given different therapies in the TC + CC genotype group, where the hormonal therapy subgroup had a longer survival than the chemotherapy subgroup (Fig. 3B). Giving chemotherapy to the TC + CC variant group brought a risk of death 2.01 times higher than giving hormonal therapy (Fig. 3B). These finding are in line with Kou’s study, which found that the ESR1 PvuII rs2234693 T/T genotype vs. C/T had a better OS when the patients were not given adjuvant chemotherapy [27]. This result is slightly different from others, which have shown that there are no specific benefits of chemotherapy or hormonal therapy in patients with circulating ESR1 exon mutated cells [6, 28].

ESR1 mutations in circulating tumour cells have been used as a predictive factor for breast cancer patients after failure of hormonal therapy [28]. One strength of the current study was genotyped ESR1 before applying the therapy to the primary tissue. The weakness of this study is that the results shown are still lacking in precision, because confidence intervals (CIs) were quite wide and the number of samples was small, so it will be necessary to investigate these issues in a larger, prospective study. Further study of the mechanisms underlying the better prognosis of patients with different genotypes PvuII rs2234693 is warranted [27].

## Conclusion

In general, TC + CC variants have a worse prognosis than TT variants. However, hormonal therapy will provide a longer survival rate than chemotherapy to the former subgroup. Our analyses provide compelling evidence that *ESR1* PvuII is a novel prognostic marker in breast cancer and is also highly predictive of anticancer therapy outcomes. It could become a predictive factor for first-line hormonal treatment outcomes because the genotype might predict which kind of therapy is expected to be more effective.

## Data Availability

The datasets for this manuscript are not publicly available because they contain personal patient information and the data belong to the Dharmais Cancer Hospital. Requests for data must be directed to [Ramadhan Karsono, ramadhan@dharmais-surgonc.com].

## Abbreviations

AI: Aromatase inhibitor
BSO: Bilateral salpingo-oophorectomy
CIs: Confidence interval
ER: Estrogen receptor
FAC: 5-fluorouracil, adriamycin, and cyclophosphamide
GnRHa: Gonadotropin-releasing hormone analogue
HRs: Hazard ratios
LBD: Ligand binding domain
NA: Not applicable
NACT: Neoadjuvant chemotherapy
NAHT: Neoadjuvant hormonal therapy
OS: Overall survival
PCR: Polymerase chain reaction
PR: Progesterone receptor
SD: Standard deviation
